# Endoscopic indicators in patients with familial adenomatous polyposis undergoing duodenal resections – a nationwide Danish cohort study with long-term follow-up

**DOI:** 10.1007/s10689-024-00415-x

**Published:** 2024-07-24

**Authors:** JG Karstensen, MD Wewer, S. Bülow, TVO Hansen, H. Højen, AM Jelsig, TP Kuhlmann, J. Burisch, HC Pommergaard

**Affiliations:** 1 Danish Polyposis Register, Gastro Unit, Copenhagen University Hospital - Amager and Hvidovre, Kettegaard Allé 30, DK-2650 Hvidovre, Denmark; 2 Department of Clinical Medicine, University of Copenhagen, Copenhagen, Denmark; 3 Gastrounit, medical division, Copenhagen University Hospital - Amager and Hvidovre, Hvidovre, Denmark; 4 Department of Clinical Genetics, Copenhagen University Hospital - Rigshospitalet, Copenhagen, Denmark; 5 Department of Pathology, Copenhagen University Hospital – Herlev and Gentofte, Herlev, Denmark; 6 Hepatic Malignancy Surgical Research Unit (HEPSURU), Department of Surgery and Transplantation, Copenhagen University Hospital - Rigshospitalet, Copenhagen, Denmark

**Keywords:** Familial adenomatous polyposis, Endoscopy, Duodenal adenomatosis, Adenocarcinoma, Genetic variants

## Abstract

Background: Familial adenomatous polyposis (FAP) predisposes individuals to duodenal adenomas. This study describes the histopathological features of endoscopic and surgical specimens from the duodenum, as well as genotype-phenotype associations. Methods: All known FAP patients were included from the Danish Polyposis Register. FAP patients were defined as having more than 100 cumulative colorectal adenomas and/or having a known germline pathogenic variant in the *APC* gene. Endoscopic procedures, histopathology, and genetics were evaluated. Results: Of 500 FAP patients, 70.6% underwent esophagogastroduodenoscopy (EGD) at least once. Of these, 59.2% presented with detectable duodenal adenomas. The most severe morphology was tubular in 62.7% patients, tubulovillous in 25.4%, and villous in 12.0%, while the most severe dysplasia was low-grade in 67.5% patients, high-grade in 25.4%, and 6.7% had adenocarcinoma. In 6.2% of FAP patients, duodenal resection was recommended, including 29% with duodenal adenocarcinoma. The risk of duodenal surgery was 1.31 per 1,000 person-years (median age: 53 years). The predominant reason for surgery was extensive polyposis (67.7%). Of the patients who underwent duodenal resection, a median of six (IQR: 4–8) EGDs were performed within five years prior to surgery, but 67.6% and 83.9% never underwent a duodenal polypectomy or endoscopic mucosa resection, respectively. Of note, seventeen of 500 patients (3.4%) developed duodenal adenocarcinoma, of which 47% were advanced at diagnosis. Genetic evaluations revealed various pathogenic variants in the *APC* gene, with no strong genotype-phenotype association. Conclusions: The prevalence of duodenal adenomas and cancer in FAP warrants vigilant endoscopic surveillance. Nevertheless, the need for duodenal surgery persists and should together with endoscopic practice be monitored in national registers.

**Supplementary Information:**

The online version contains supplementary material available at 10.1007/s10689-024-00415-x.

## Introduction

Familial adenomatous polyposis (FAP) is an autosomal dominantly inherited disorder, which in addition to colorectal polyposis predisposes individuals to duodenal adenomatosis and cancer [1–5]. In the twentieth century, prophylactic colectomy was introduced for FAP patients and it decreased the risk of colorectal cancer and resulted in prolonged life expectancy [6]. Consequently, the importance of duodenal manifestations has increased. To prevent the development of duodenal cancer, it is essential to identify high-risk cases early on and refer them for surgical intervention before they undergo malignant transformation. Presently, the Spigelman classification system offers a comprehensive approach for endoscopic staging of duodenal adenomatosis and for assessing the risk of duodenal cancer. This system integrates factors such as the number and size of the adenomas, along with their morphology and the extent of dysplasia [7]. Although the Spigelman classification has been validated, it tends to underestimate the importance of ampullary lesions and does not closely correlate with the risk of duodenal cancer [8–11]. Additionally, it requires obtaining biopsies from duodenal lesions that many endoscopists would prefer to remove completely, either by simple polypectomy or endoscopic mucosal resection (EMR) [12–14].

Duodenal resection is indicated in the event of a localized duodenal cancer, as well as prophylactically in cases with severe polyposis and/or an assumed high risk of cancer where endoscopic surveillance and treatment is considered insufficient [15, 16]. However, the threshold for duodenal resection is not clearly defined and the need for prophylactic surgical resection might be reduced with increased use of invasive endoscopic techniques.

In Denmark, all known patients with FAP have been meticulously registered in the Danish Polyposis Register for the last 40 years, which includes data about endoscopic and surgical procedures, as well as genetic reports [17]. The register is free of referral and selection bias, which helps to ensure reliable estimations for the risk of needing duodenal surgery, as well as preoperative endoscopic interventions and other risk factors.

We evaluated the histopathological severity of duodenal polyposis in the surgical specimens and compared it with previous endoscopic examinations, as well as genotypes. Furthermore, we examined whether the need for duodenal resections has been reduced in recent decades, possibly as a benefit of endoscopic interventions.

## Methods

The Danish Polyposis Register was established in 1971 and became nationwide in 1974 [17]. It comprises all Danish FAP patients. Endoscopic, surgical, and histopathological reports are all included, together with pedigrees and genetic test results. We conducted a cohort study of all known patients with FAP. No ethics approval or informed written consent were needed as this was a cohort study.

### Definitions

FAP patients were defined as having 100 cumulative colorectal adenomas or more and/or having a known germline pathogenic variant in the *APC* gene (pathogenic or likely pathogenic, using American College of Medical Genetics and Genomics/the Association for Molecular Pathology *APC* gene-specific guidelines) [18]. Patients with more than 100 colorectal adenomas and a known non-*APC-*related genetic etiology were excluded from the register and this study.

### FAP cohort

The cohort consisted of all verified FAP patients registered in the Danish Polyposis Register up until April 22nd, 2021. Patients needed to have been alive on January 1st 1990 and should not have undergone duodenal surgery or developed duodenal cancer before initiation of the study. Patients with a duodenal resection (Whipple procedure or total pancreatectomy) due to pancreatic premalignant lesions or cancer were excluded. Since 1968, all Danish individuals have had a unique, 10-digit personal identification number [19]. We submitted the identification numbers of the FAP patients to Statistics Denmark, which enabled us to extract a complete list of endoscopic and surgical procedures from the National Patient Register (Supplementary Material 1), alongside the histopathological results from the Danish Pathology Register (Supplementary Material 2). Additionally, genotypes and indications for surgery were provided by the Polyposis Register.

### Outcomes

The primary outcome was duodenal resection due to duodenal adenomatosis or cancer defined as risk per 1,000 person-years. Pancreatic indications were excluded. Secondary outcomes included the risk of developing duodenal adenomas and their morphology and grade of dysplasia. Additionally, the associations between surgical and endoscopic findings, in terms of adenoma morphology, grade of dysplasia, and adenocarcinoma, were analyzed, together with the risk of duodenal surgery. The most severe morphology and grade of dysplasia in each patient were counted. The most severe morphology was defined as villous, followed by tubulovillous, then tubular. Surgical and endoscopic modalities for the treatment of duodenal adenomas were assessed. Finally, the genotypes for all patients with duodenal resections were noted. According to the regulations of Statistics Denmark, absolute numbers of groups smaller than three were omitted.

### Statistical methods

Follow-up of patients started on the date of their FAP diagnosis or on January 1st, 1990, whichever was most recent. Follow-up ended on the date of duodenal resection, death, loss to follow-up, or the end of the study, whichever occurred first. Baseline characteristics of the cohort were described using medians and interquartile ranges (IQR) for numerical variables and counts and proportions for categorical variables. A two-sided *P* value < 0.05 was considered significant. *R* version 4.2.1 (R Foundation for Statistical Computing, Vienna, Austria) was used to perform all statistical analyses.

## Results

### Characteristics of patients with familial adenomatous polyposis

The cohort comprised 500 eligible FAP patients; 235 were female (47%) (Table 1). The genotype was known in 439 (87.8%) patients, and 176 were probands (35%). Of the 500 patients, 17 (3.4%) developed duodenal cancer (adenocarcinoma), 14 of which were identified in biopsies taken during an esophagogastroduodenoscopy (EGD). The remaining three cases of adenocarcinomas were found in the resected specimens in patients with high-grade dysplasia (HGD) in the endoscopic biopsies prior to surgery.

**Table 1:** Danish nationwide cohort of familial adenomatous polyposis cohort characteristics

Cohort characteristics	FAP patients, *N* = 324
Female	235 (47%)
Year of birth	
< 1920	3 (0.6%)
1920–1930	16 (3.2%)
1930–1940	37 (7.4%)
1940–1950	54 (10.8%)
1950–1960	71 (14.2%)
1960–1970	89 (17.8%)
1970–1980	70 (14.0%)
1980–1990	60 (12.0%)
1990–2000	60 (12.0%)
> 2000	40 (8.0%)
Probands	176 (35.2%)
Genotype	
Yes	439 (87.8%)
No	61 (12.2%)
Number of patients with duodenal surgery	31 (6.2%)
Median (IQR) time to duodenal surgery (years)	52 (40, 59)

FAP, familial adenomatous polyposis; IQR, inter quartile range

### Endoscopy

**Fig. 1:**
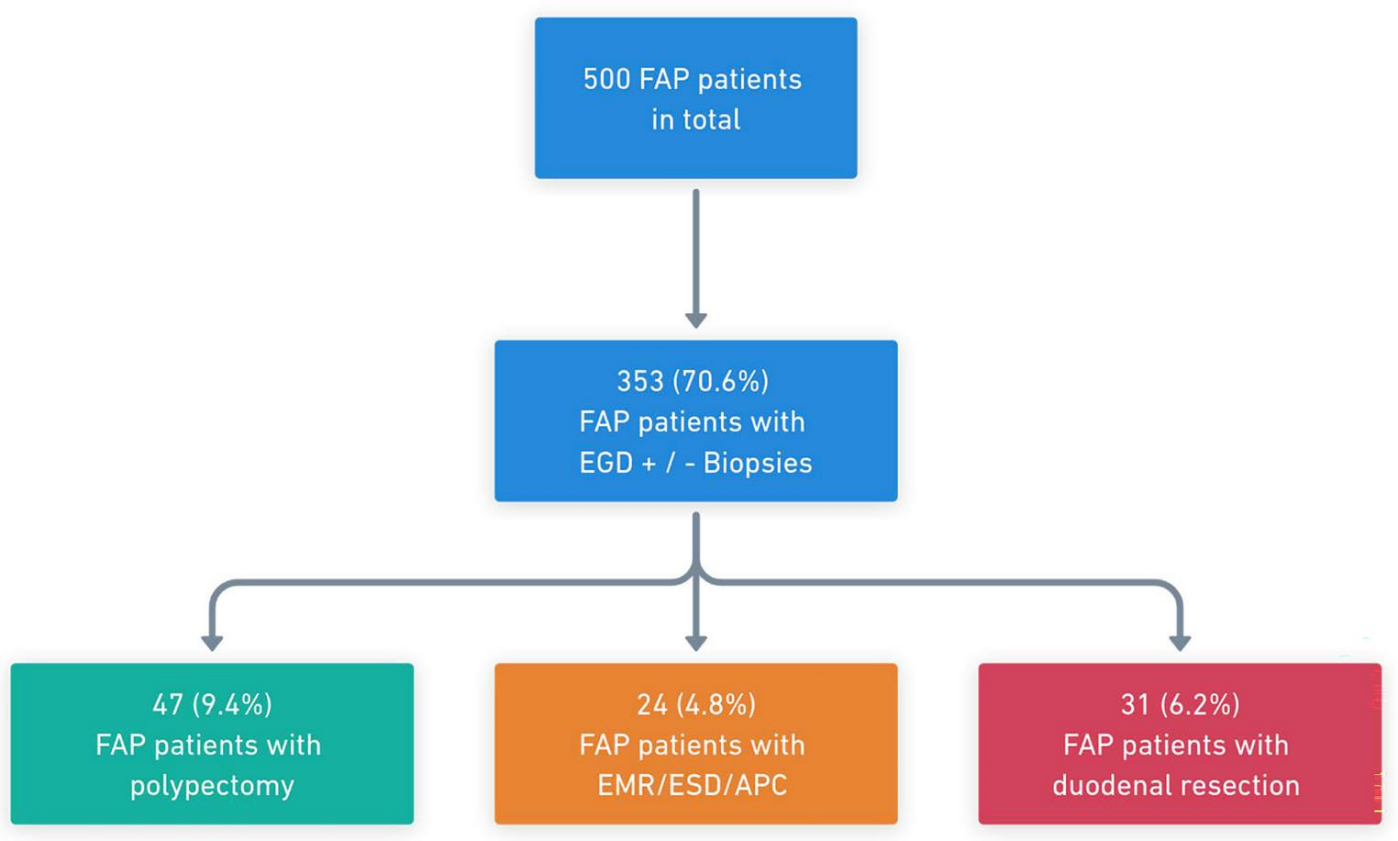
Flow chart for a Danish nationwide cohort of patients with familial adenomatous polyposis *FAP: familial adenomatous polyposis; EGD: esophagogastroduodenoscopy; EMR: endoscopic mucosal resection; ESD: endoscopic submucosal dissection; APC: argon plasma coagulation

During the follow-up period, 60.8% (304/500) of patients received at least one EGD, 53.0% (265/500) an EGD with biopsies of duodenal polyps, and 70.6% (353/500) either at least one EGD or an EGD with biopsies. Of those who did not receive an EDG, two out of three were either below the age where duodenal surveillance is initiated or died due to CRC before initiating duodenal surveillance. Duodenal polypectomy was performed in 9.4% of the FAP patients (47/500), while 4.8% (24/500) underwent endoscopic mucosal resection (EMR)/endoscopic submucosal dissection (ESD)/argon plasma coagulation of duodenal lesions (Fig. 1).

**Fig. 2:**
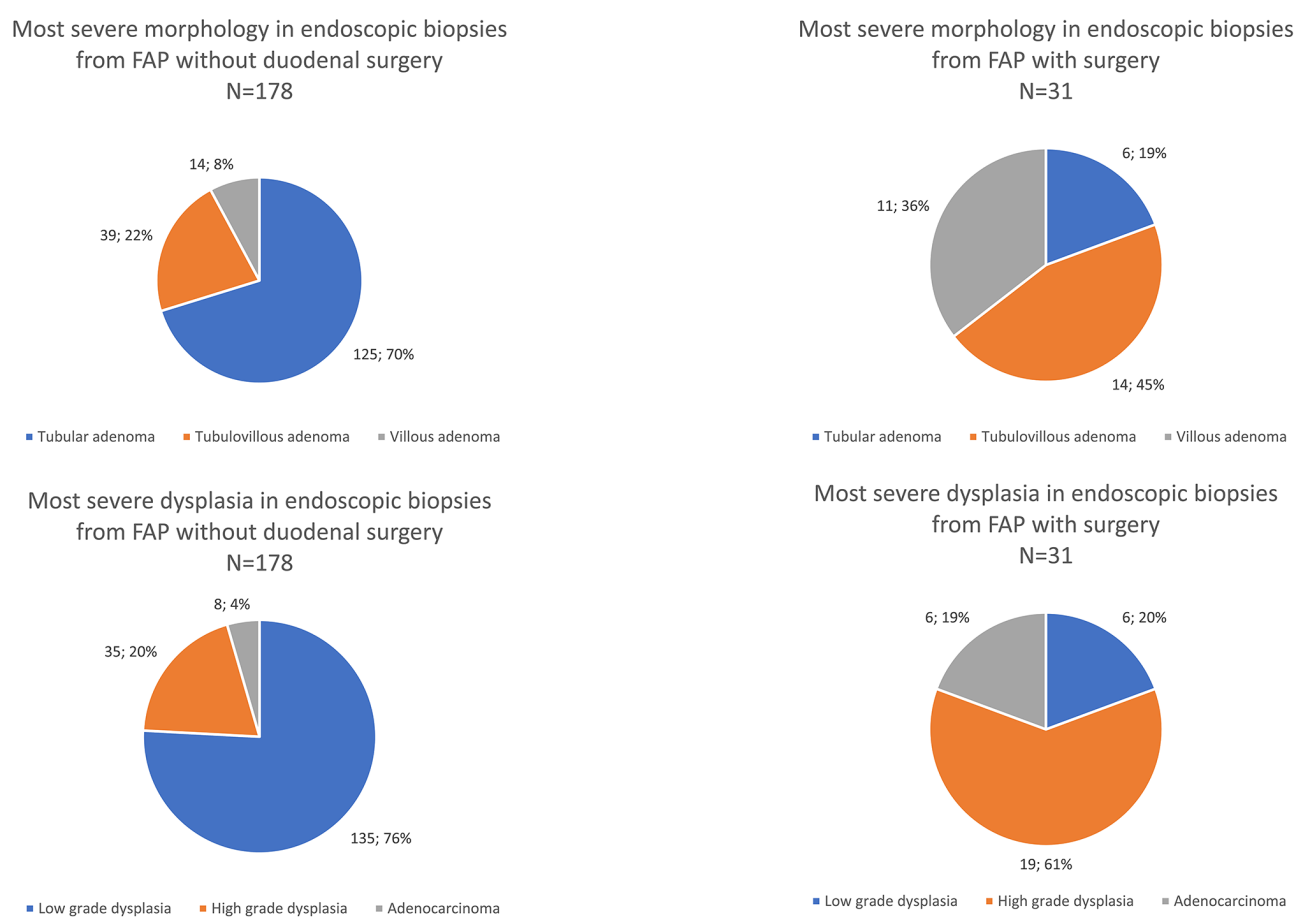
Distribution of the most severe morphologies and dysplasia in familial adenomatous polyposis patients with endoscopic biopsies prior to potential surgery. *FAP: familial adenomatous polyposis

In 59.2% (209/353) of patients who underwent at least one EGD, the histopathology from either an endoscopic biopsy or a resection showed adenoma. The most severe morphology in these patients included 62.7% (131/209) with tubular adenomas, 25.4% (53/209) with tubulovillous adenomas, and 12.0% (25/209) with villous adenomas (Fig. 2). There was low-grade dysplasia (LGD) in 67.5% (141/209) and HGD in 25.4% (53/209) of patients (Fig. 2). The histopathological diagnosis was adenocarcinoma in 3.9% of FAP patients who received at least one EGD (14/353), corresponding to 6.7% of patients with known duodenal adenomatosis (14/209). Of the 14 patients with malignant histopathology, 57.1% (8/14) did not undergo surgery due to disseminated disease.

### Duodenal surgery

During the follow-up period, 6.2% (31/500) of FAP patients underwent duodenal resection, corresponding to a risk of 1.31 per 1,000 person-years. The median age at surgery was 53 years (IQR = 41–62 years) and 39% (12/31) of patients were female. A Whipple procedure was performed in 67.7% (21/31), while the remaining patients underwent a pancreas-preserving duodenectomy. The histopathology in the resected specimens included adenocarcinoma in 29% (9/31) of the cases and benign histology in the remaining 22 cases (71%). In three of the nine patients diagnosed with adenocarcinoma, the endoscopic biopsies prior to surgery showed HGD as the most severe morphology. However, upon surgical resection, adenocarcinoma was identified in the specimens. In all benign cases, the histopathology showed adenomas with HGD in 86.3% (19/22) of cases and LGD in 13.6% (3/22) of cases. Over a median follow-up period of 9 years, no patients who underwent a duodenectomy required conversion to a Whipple procedure.

### Indication for duodenal surgery

The indication for duodenal resection was extensive duodenal adenomatosis prohibiting safe endoscopic surveillance or treatment in 67.7% (21/31) of patients. In the remaining cases, there was an endoscopic suspicion of either an ampullary (22.6%) or luminal (6.5%) cancer. Of these cases, the suspicion of malignancy was confirmed in 88.9% by histopathological examination of the surgical specimen. The indication was not clear in 3.2% (1/22) of cases. For patients receiving surgery with the indication of extensive polyposis, 9.5% (2/21) were operated upon between 1990 and 1999, 38.1% (8/21) between 2000 and 2009, and 52.3% (11/21) between 2010 and 2019.

### Genetics

**Fig. 3:**
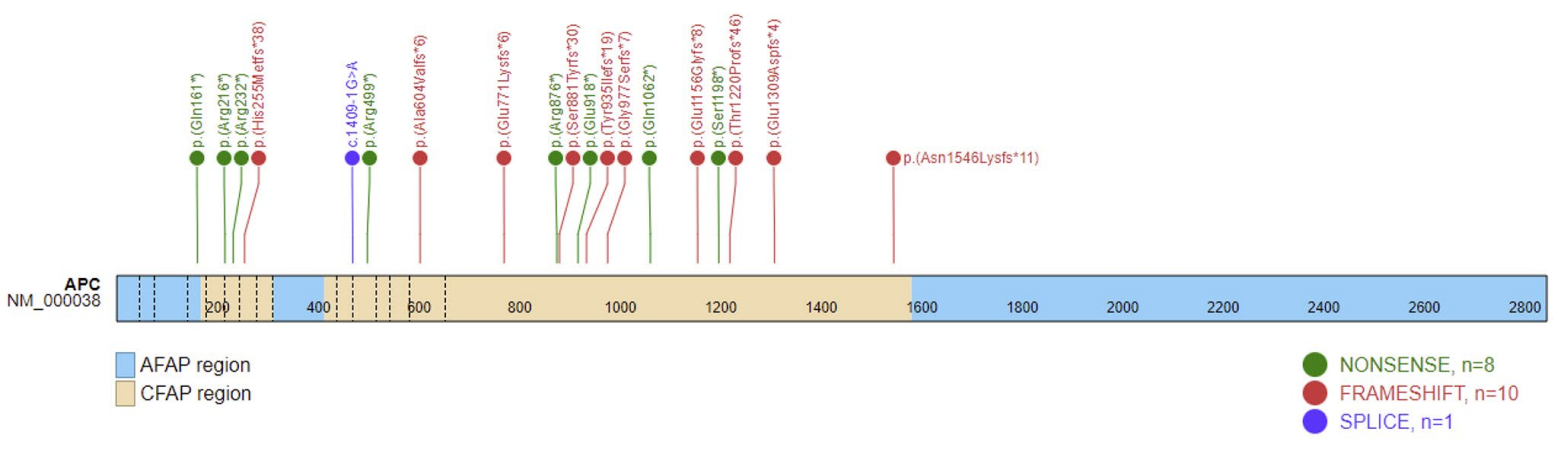
Lollipop plot showing the *APC* (NM_000038.6) single nucleotide variants described in this study in relation to attenuated FAP (AFAP) (light blue) and classical FAP (CFAP) (light brown) regions of the *APC* gene. The variants include 10 frameshift variants (red), eight nonsense variants (green) and one splice variant (blue). Only one variant (p.(Gln161*)) is located in the AFAP region. Exons are indicated with dashed lines starting with the first coding exon (exon 2)

Patients undergoing duodenal resection or developing unresectable duodenal cancer comprised 28 families; the pathogenic variant was known in 89.7% (35/39) of patients. Three variants (p.(Glu1309Aspfs*4), p.(Glu1156Glyfs*8) and p.(Gln161*)) were detected in more than one family, but otherwise each family carried a different variant. All pathogenic variants were frameshift, nonsense, or splice variants, including single nucleotide variants, smaller or larger deletions or duplications, or large rearrangements (including whole *APC* gene deletion in one family) (Fig. 3). A need for duodenal resection or unresectable duodenal cancer was identified in six patients from one family who were carrying the c.2626C > T, p.(Arg876*) variant. Two families had a variant that was located 5’ of codon 168 in an area associated with A-FAP, while no variants were detected in other A-FAP regions in the *APC* gene, including codon 312–412 (alternative part of exon 9) and 3’ of codon 1580 (Fig. 3). Additionally, one family had a variant in the codon 976–1067 region. Most pathogenic variants were localized in exon 16. We failed to identify any firm genotype-phenotype correlation.

### Endoscopic findings prior to surgery

Prior to the surgical procedure, all patients had received at least one EGD. Of the nine patients with adenocarcinoma in their surgical specimen, 66.7% (6/9) had endoscopic biopsies with adenocarcinoma, while 33.3% (3/9) had HGD in the endoscopic biopsies prior to surgery. In cases with a benign surgical pathology, endoscopic biopsies or polypectomy/EMR specimens before surgery included LGD in 27.3% (6/22) of cases and HGD in 72.7% (16/22) of cases (Table 2). A median of six (IQR: 4–8) EGDs were performed within the five years prior to surgery and 35.5% (11/31) had villous adenomas (Table 2). In 67.6% (21/31) of patients who received a duodenal resection, the patient never underwent a duodenal polypectomy and EMR/ESD/APC were not carried out in 83.9% (26/31) of patients.

**Table 2:** Most severe endoscopic morphology and dysplasia of duodenal adenomas in familial adenomatous polyposis patients prior to potential surgery

Specific pathology	FAP patients without duodenal surgery *N* = 469		FAP patients with duodenal surgery *N* = 31		*p-values
**Morphology**					< 0.001
Tubular adenoma	125	(26.7%)	6	(19.4%)	
Tubulovillous adenoma	39	(8.3%)	14	(45.2%)	
Villous adenoma	14	(3.0%)	11	(35.5%)	
**Dysplasia**					< 0.001
Low-grade dysplasia	135	(28.8%)	6	(19.4%)	
High-grade dysplasia	35	(7.5%)	19^1^	(61.3%)	
Adenocarcinoma	8	(1.7%)	6	(19.4%)	

FAP: familial adenomatous polyposis

*Fisher’s exact test ^1^Three patients undergoing duodenal surgery had adenocarcinoma in the surgical specimen and high-grade dysplasia in the endoscopic biopsies

## Discussion

In this nationwide cohort study of all known Danish FAP patients, we found that during a 30-year period the risk of duodenal surgery was 1.31 per 1,000 person-years with a median age at surgery of 53, and an increasing number of resections being carried out during this period. In 71.0% of FAP patients undergoing duodenal surgery, the indications, as well as the final histopathology, were benign. However, two-out-of-three patients never underwent a duodenal polypectomy before surgery, and only 16% had a duodenal EMR, thus emphasizing that the full potential of endoscopic interventions might not have been thoroughly explored. This also indicates that the described risk of duodenal surgery closely follows the natural progression of developing duodenal adenomatosis over time.

Studies have reported a lifetime risk of duodenal adenomatosis in up to 90% of individuals with FAP [2]. The progression from adenoma to adenocarcinoma in the duodenum, albeit slower than in the colon and rectum, remains a significant cause of morbidity and mortality [3, 6]. A recent study demonstrated that FAP patients had a 14-fold higher risk of developing duodenal/small bowel cancer compared to the general population [1]. Thus, regular surveillance for duodenal lesions is paramount. In addition to surveillance, a growing body of evidence suggests that endoscopic techniques can obviate the need for surgery in a significant proportion of patients [13, 20, 21]. However, challenges remain. While EMR is efficient in removing larger lesions, duodenal EMR has its own set of adverse events such as bleeding, perforation, and post-polypectomy syndrome. Nevertheless, recent studies evaluating the use of cold snares for EMR have shown promising results, with fewer adverse events and few recurrent lesions [22–24]. Likewise, duodenal polypectomy, either with hot or cold snares, seems very safe and might remove duodenal lesions before they advance [13, 25]. In our study, we found that only a minority of patients had undergone endoscopic removal of duodenal lesions before surgery. While our study’s analyses cannot definitively determine if some surgeries could have been avoided, the data strongly suggest that most patients did not receive the full benefit of currently available endoscopic therapies and the potential of endoscopic therapy was not integrated in the surveillance of these patients. This is despite of centralized surveillance in four centers for three decades. A further centralization into two centers each covering around 200 patients might be ideal to facilitate dedicated patient care.

Endoscopic techniques, while reducing the need for surgery, cannot always replace it, especially for ampullary lesions extending into the pancreatic or common bile duct. In FAP patients with duodenal lesions, choices often oscillate between the Whipple procedure, known for its comprehensive resection and associated complications, and the less invasive pancreas-preserving duodenectomy. The latter, while preserving pancreatic function, can raise the risk of recurrence and limit lymph node clearance in cases of malignancy [26]. Our study showed that two-thirds of patients underwent a Whipple procedure, probably reflecting the presence or suspicion of a malignant lesion. Notably, while the number of Whipple procedures seems to be on the rise, there is a declining trend in pancreas-preserving duodenectomies. This may complicate post-operative endoscopic management, as deep small bowel enteroscopy is needed to inspect the Roux-en-Y limb because of the Whipple operation. The cause of this trend remains undetermined. It might be influenced by surgical preferences, or the future risk of requiring a Whipple procedure due to ampullary adenomatosis [26].

The FAP patients who received a duodenal resection, together with those who developed disseminated duodenal cancer, represent the most severe phenotype. We analyzed the pathogenic *APC* variants in all these patients and found that only one family had a variant in the codon 976–1067, which has previously been associated with a 3-4-fold risk of developing duodenal adenomatosis [27]. Furthermore, one variant identified in two families was somewhat surprisingly located in an area of the gene which has previously been associated with a less severe phenotype (attenuated FAP) [28–30]. We find that the number of families/patients were too few for us to conclude there is a firm phenotype-genotype correlation; hence, endoscopic surveillance and treatment cannot be stratified according to specific pathogenic variants in the *APC* gene based on the present data. Nonetheless, we identified 6 cases of duodenal resections/unresectable cancer in one family who was carrying the c.2626 C > T, p.(Arg876*) pathogenetic variant and in this family, intensified surveillance might be justified.

The evolving role of endoscopic interventions, particularly polypectomy, EMR and endoscopic papillectomy in managing duodenal lesions in FAP cannot be understated [20, 31–33]. While they offer significant advantages over surgical modalities, a comprehensive, individualized approach is crucial to ensure optimal patient outcomes [12]. Further studies, preferably comparative, that focus on long-term outcomes and newer endoscopic techniques, are eagerly awaited. Of note, in our study the number of endoscopic resections was limited, hence, the FAP cohort may be considered representing the long-term natural course of duodenal adenomatosis under endoscopic surveillance.

This study is limited by the small number of patients undergoing duodenal resection. Furthermore, our knowledge of the endoscopic surveillance before referral for surgery is limited to procedural codes and details such as Spigelman classification and possible reasons for omitting duodenal surveillance are not available. Likewise, we cannot fully explain why half of the patients with duodenal cancer were disseminated at the time of diagnosis. However, the study’s strengths include a national database free of referral and selection bias, as well as access to pathology reports after both endoscopy and surgery for comparison. We have not included adverse events of endoscopic and surgical interventions as we do not find the coding consistent over the complete study period. Finally, endoscopic technology has been improved considerably during the study period, which might have improved the optical diagnoses.

Our nationwide cohort study encompassing the entire Danish FAP population revealed a risk of duodenal surgery of 1.31 per 1,000 person-years, with patients undergoing surgery at a median age of 53 years. Strikingly, two-thirds of the patients referred for surgical intervention had not previously received a duodenal polypectomy, and even fewer an EMR. Furthermore, most patients were found to have a benign histopathology in their surgical specimen. To ensure the quality of endoscopic surveillance and interventions, we find it essential to monitor endoscopic practice and development of duodenal cancer in national registers.

## Electronic supplementary material

Below is the link to the electronic supplementary material.

Supplementary Material 1

## Data Availability

Anonymized and summarized data collected for the study will be made available to other researchers upon publication and following reasonable requests made to the corresponding author.
