# Supplementary material for: Endoscopic indicators in patients with familial adenomatous polyposis undergoing duodenal resections – a nationwide Danish cohort study with long-term follow-up
Source: Fam Cancer. 2024 Jul 24;23(4):607–15. doi: 10.1007/s10689-024-00415-x (PMC11512927; doi:10.1007/s10689-024-00415-x)
Supplement: Supplementary file 1 — Supplementary Material 1 [file 10689_2024_415_MOESM1_ESM.docx]

# Supplementary material

**Supplementary Table 1. Surgical procedures**

| **Intestinal surgery** | **Danish classification of surgical procedures and therapies (1977-1995)** | **Danish classification of surgical procedures and therapies (1996-)** |
| --- | --- | --- |
| Duodenum | 42240, 43120, 43160, 43170, 43180, 43200, 43220, 43240, 43314, 44580, 46080, 46250, 47200, 47300, 47800, 47801, 48550, 48620, 48760, | KJDH |
| **Endoscopy** |  |  |
| Esophagogastroduodenoscopy (EGD) | 93170, 91010 | KUJD |

**Supplementary Table 2. SNOMED histopathology codes**

Duodenal T-codes:

| T58700 | Papilla and ampulla of Vater |
| --- | --- |
| T587A0 | Periampullary region |
| T64391 | Major duodenal papilla |
| T64396 | Major duodenal papilla mucosa |
| T64400 | Hepatopancreatic ampulla (of Vater) |

| T64310 | Duodenal mucosa |
| --- | --- |
| T64311 | Duodenal mucosa, bulbus |
| T64312 | Duodenal mucosa, 2nd segment |
| T64391 | Major duodenal papilla |
| T64396 | Major duodenal papilla mucosa |
| T64400 | Hepatopancreatic ampulla (of Vater) |
| T64950 | Duodenum and jejunum |
| T64300 | Duodenum |

| M-codes: |  |
| --- | --- |
| M82110 | Tubular adenoma |
| M82611 | Villous adenoma |
| M82630 | Tubulovillous adenoma |

| M81482 | High-grade neoplasia |
| --- | --- |
| M74C09 | High-grade dysplasia |
| M74HG9 | High-grade dysplasia |
| M74A09 | Low-grade dysplasia |
| M74LG9 | Low-grade dysplasia |
| M814A0 | Low-grade neoplasia |
| M81403 | Adenocarcinoma |
| M82103 | Adenocarcinoma in a polyp (polyp cancer) |
| M82313 | Poorly differentiated adenocarcinoma |
| M84803 | Mucinous adenocarcinoma |

T-codes are for topography (localization) and M-codes are for morphology (histopathology)
